# RASflow: an RNA-Seq analysis workflow with Snakemake

**DOI:** 10.1186/s12859-020-3433-x

**Published:** 2020-03-18

**Authors:** Xiaokang Zhang, Inge Jonassen

**Affiliations:** Computational Biology Unit, Department of Informatics, University of Bergen, Thormohlens Gate 55, Bergen, 5009 Norway

**Keywords:** RNA-Seq, Workflow, Snakemake

## Abstract

**Background:**

With the cost of DNA sequencing decreasing, increasing amounts of RNA-Seq data are being generated giving novel insight into gene expression and regulation. Prior to analysis of gene expression, the RNA-Seq data has to be processed through a number of steps resulting in a quantification of expression of each gene/transcript in each of the analyzed samples. A number of workflows are available to help researchers perform these steps on their own data, or on public data to take advantage of novel software or reference data in data re-analysis. However, many of the existing workflows are limited to specific types of studies. We therefore aimed to develop a maximally general workflow, applicable to a wide range of data and analysis approaches and at the same time support research on both model and non-model organisms. Furthermore, we aimed to make the workflow usable also for users with limited programming skills.

**Results:**

Utilizing the workflow management system Snakemake and the package management system Conda, we have developed a modular, flexible and user-friendly RNA-Seq analysis workflow: RNA-Seq Analysis Snakemake Workflow (RASflow). Utilizing Snakemake and Conda alleviates challenges with library dependencies and version conflicts and also supports reproducibility. To be applicable for a wide variety of applications, RASflow supports the mapping of reads to both genomic and transcriptomic assemblies. RASflow has a broad range of potential users: it can be applied by researchers interested in any organism and since it requires no programming skills, it can be used by researchers with different backgrounds. The source code of RASflow is available on GitHub: https://github.com/zhxiaokang/RASflow.

**Conclusions:**

RASflow is a simple and reliable RNA-Seq analysis workflow covering many use cases.

## Background

RNA sequencing (RNA-Seq) was introduced more than ten years ago and has become one of the most important tools to map and identify genes and understand their regulation and roles across species [1, 2]. A large number of studies have been performed using RNA-Seq and resulted in gene expression datasets available in databases such as GEO [3] and ArrayExpress [4]. Underlying reads are typically deposited to the Sequence Read Archive (SRA) [5], currently containing reads for more than 1,7 million samples (https://www.ncbi.nlm.nih.gov/sra/?term=RNA-Seq). One of the most popular applications of RNA-Seq is for Differential Expression Analysis (DEA) where one identifies genes that are expressed at different levels between two classes of samples (e.g., healthy, disease) [6].

When RNA-Seq is used in a DEA project, the sequencing reads need to be taken through several steps of processing and analysis. Often, the steps are organized into a workflow that can be executed in a fully or partially automated fashion. The steps include: quality control (QC) and trimming, mapping of reads to a reference genome (or transcriptome), quantification on gene (or transcript) level, statistical analysis of expression statistics to report genes (or transcripts) being differentially expressed between two predefined sets of samples, along with associated *P*-values or False Discovery Rate (FDR) values. Aligning reads to the genome is the most computationally intensive and time-consuming step. An alternative approach is to perform a pseudo alignment to a transcriptome. This has gained more popularity recently, due to its high speed and high accuracy [7–9]. It has been shown that lightweight pseudo alignment improves gene expression estimation and at the same time is computationally more efficient, compared with the standard alignment/counting methods [10]. But if the purpose of analysis is to call genomic variants, then it is still better to map the reads to the genome [11]. Considering this, a workflow should provide both quantification strategies to satisfy users with different research interests.

There is a large number of RNA-Seq analysis workflows and many have been published and made available to the user community. We reviewed seven workflows published in the past three years [12–18] (see “Discussion” section for more details). We found that none of these workflows cover all the needs outlined above while also being usable for less computer fluent users. So more complete and easy-to-use workflows are still needed.

In this article, we present RNA-Seq Analysis Snakemake Workflow (RASflow) that is usable for a wide range of applications. RASflow can be applied to data from any organism and can map reads to either a genome or a transcriptome, allowing the user to refer to public databases such as ENSEMBL [19] or to supply their own genomes or transcriptomes [20, 21]. The latter can for example be useful for projects on non-model species for which there is no public high-quality reference genome/transcriptome. RASflow is scalable: it can be run on either supercomputers with many cores (which enable parallel computing) or on a personal computer with limited computing resources; it can process data from hundreds of samples and still consumes very little storage space because it temporarily copies or downloads the FASTQ file(s) of one sample (one file for single end and two files for pair end) to the working directory at the time, and it stores only the necessary intermediate and final outputs. Using Conda [22], the whole workflow with all dependencies (version already specified) can be installed simply with one single command in a virtual environment. This ensures quick and smooth installation. Using Snakemake [23], the whole analysis is completely reproducible and highly user-friendly also for users with limited programming skills. In the DEA step, RASflow supports use of paired tests that can help to strengthen the statistical power and bring out expression differences related to the phenomenon under study [24].

## Implementation

**Fig. 1:**
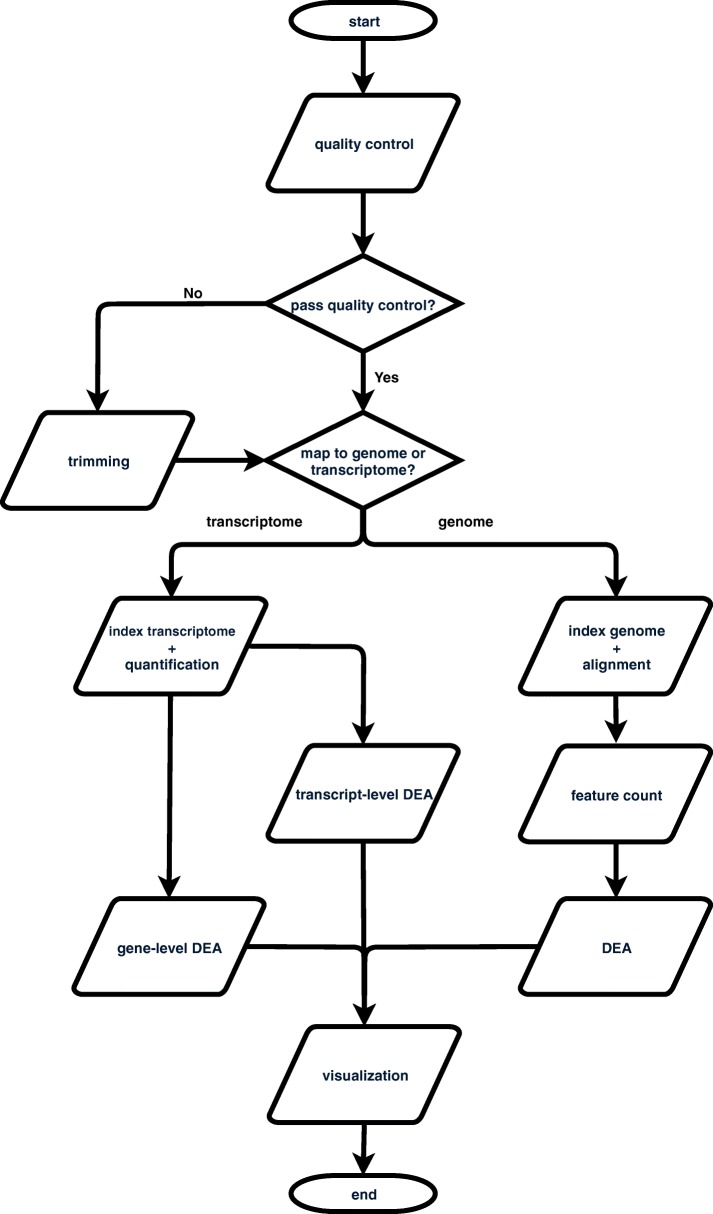
Overview of the steps performed by RNA-Seq Analysis Snakemake Workflow (RASflow)

Figure 1 shows a schematic representation of the RASflow workflow. It starts with performing QC of the raw FASTQ files using FastQC (https://www.bioinformatics.babraham.ac.uk/projects/fastqc/). The QC report is presented to the user along with a question of whether the reads should be trimmed. When opted for, trimming is performed using the tool Trim Galore (https://www.bioinformatics.babraham.ac.uk/projects/trim_galore/) and subsequently, an additional QC report is generated.

When the user is satisfied with the quality of the reads, the workflow proceeds to the next step: quantification of read abundance or expression level for transcripts or genes. The user decides whether to map the reads to a transcriptome or a genome depending on the goal of the analysis and availability of data. If the purpose of the analysis is to identify differentially expressed genes, it is suggested to map the reads to a transcriptome using pseudo alignment with Salmon [9]. A quantification table of the transcripts is generated from this step. Alternatively, the user can choose for the reads to be mapped to a genome. The aligner used in RASflow is HISAT2 [25] which has relatively modest memory requirements (∼4.3GB for the human genome) compared with for example the STAR aligner (requiring ∼27GB for the human genome) [26]. The alignment step is followed by a quality evaluation performed by Qualimap2 [27] and feature counting done by featureCounts [28] or htseq-count [29]. To be noted, after most of the steps, a summary report is generated using MultiQC [30].

When a quantification matrix for the genes/transcripts has been produced, RASflow can proceed to perform a DEA analysis using edgeR [31, 32] or DESeq2 [33]. RASflow supports both single and paired statistical tests. The user specifies which statistical test mode to be applied in the configuration file based on their experimental design. If the reads were mapped to a transcriptome, DEA will be done on both transcript- and gene-level. In any case, the outputs of DEA include three types of tables: normalized quantification tables, some important statistics for the whole gene or transcript list, and the list of significantly differentially expressed genes or transcripts (with default threshold of *FDR*<0.05). The raw count is normalized based on Trimmed Mean of M values (TMM) [34] (if edgeR is used) or the median-of-ratios method [35] (if DESeq2 is used) when the reads are mapped to a genome. But if the reads are mapped to a transcriptome, the normalized values are estimated Transcripts Per Million (TPM) from Salmon scaled using the average transcript length over samples and then the library size by "tximport" [36]. The results of DEA is also visualized with a volcano plot enabling visual identification of genes with high fold change whose differential expression is also statistically significant, and a heatmap that not only visualizes the expression pattern of the identified differentially expressed genes, but also a clustering of the samples based on those genes, so that the user can get an idea of how well separated the groups are.

To ensure smooth installation and reproducibility of the workflow, all the tools included are fixed to a specific version which can be found in the environment configuration file (env.yaml).

## Results

To show users how RASflow works and to familiarize them with RASflow, we provide some small example datasets. They are generated as subsets of the original real data [37]. The figures in this section were generated by RASflow using the example data as input. RASflow was also tested on four real datasets: pair-end RNA-seq of prostate cancer and adjacent normal tissues from 14 patients (ArrayExpress accession: E-MTAB-567) [38], single-end RNA-Seq of mesenchymal stem cells (MSCs) and cancer-associated fibroblasts (CAFs) from EG7 tumor-bearing mice (GEO accession: GSE141199), pair-end RNA-Seq of Atlantic cod liver slices exposed to benzo[a]pyrene (BaP) and 17 *α*-ethynylestradiol (EE2) (GEO accession: GSE106968) [39], and a benchmarking dataset, single-end RNA-Seq of highly purified human classical and nonclassical monocyte subsets from a clinical cohort (SRA accession: SRP082682) [40].

The output of the example dataset can be found on the GitHub page of RASflow and an overview of the output folder is shown in Additional file 1: Fig. S1. The output of the four real datasets can be found here: https://git.app.uib.no/Xiaokang.Zhang/rasflow_realdata.

### Quality control of raw reads and alignments

**Fig. 2:**
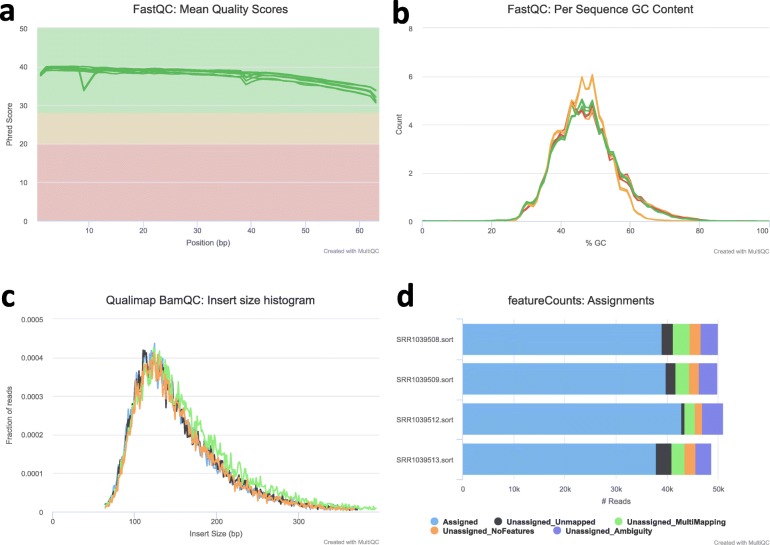
Quality control of raw reads and alignment. **a** The mean quality value across each base position in the read. **b** The average GC content of reads. A normal random library typically has a roughly normal distribution of GC content. **c** Distribution of estimated insert sizes of mapped reads. **d** A brief mapping summary

FastQC checks the quality of the sequencing reads and produces one report for each FASTQ file. MultiQC is used to summarize all the reports and merge them into one document, as shown in Fig. 2a and b. Users are asked to check the report and decide whether trimming is needed. If the quality of the reads is good enough, it is recommended that trimming should not be performed since it would lead to loss of information; but if the quality is low, trimming is suggested to improve the quality. The raw reads quality of the human prostate dataset is not good enough and trimming was therefore performed. The QC reports of raw reads and trimmed reads can be found in Additional file 2: Fig. S2.

After the alignment to the genome, the intermediate output, the BAM files, will be provided to Qualimap2 to evaluate the alignment quality. Figure 2c shows an example report from Qualimap2.

MultiQC is used to generate a report on the mapping ratios using the output of feature counting (Fig. 2d).

### Quantification of transcripts or genes

If a transcriptome was used as mapping reference, a file containing the estimated relative abundance and length of the target transcript is generated for each sample. If the reads were aligned to a genome, the direct outputs from alignment are genes’ raw count tables for each sample.

### Differential expression analysis

In the first step, the user-specified information on sample groups is used to produce one count or abundance file for each group. The raw count or abundance in those files is then normalized by either edgeR or DESeq2 generating a corresponding file for each of them. When a transcriptome is used as mapping reference, depending on user parameters, gene-level raw and normalized abundance can also be generated, and the downstream DEA will also be done on both transcript- and gene-level.

**Fig. 3:**
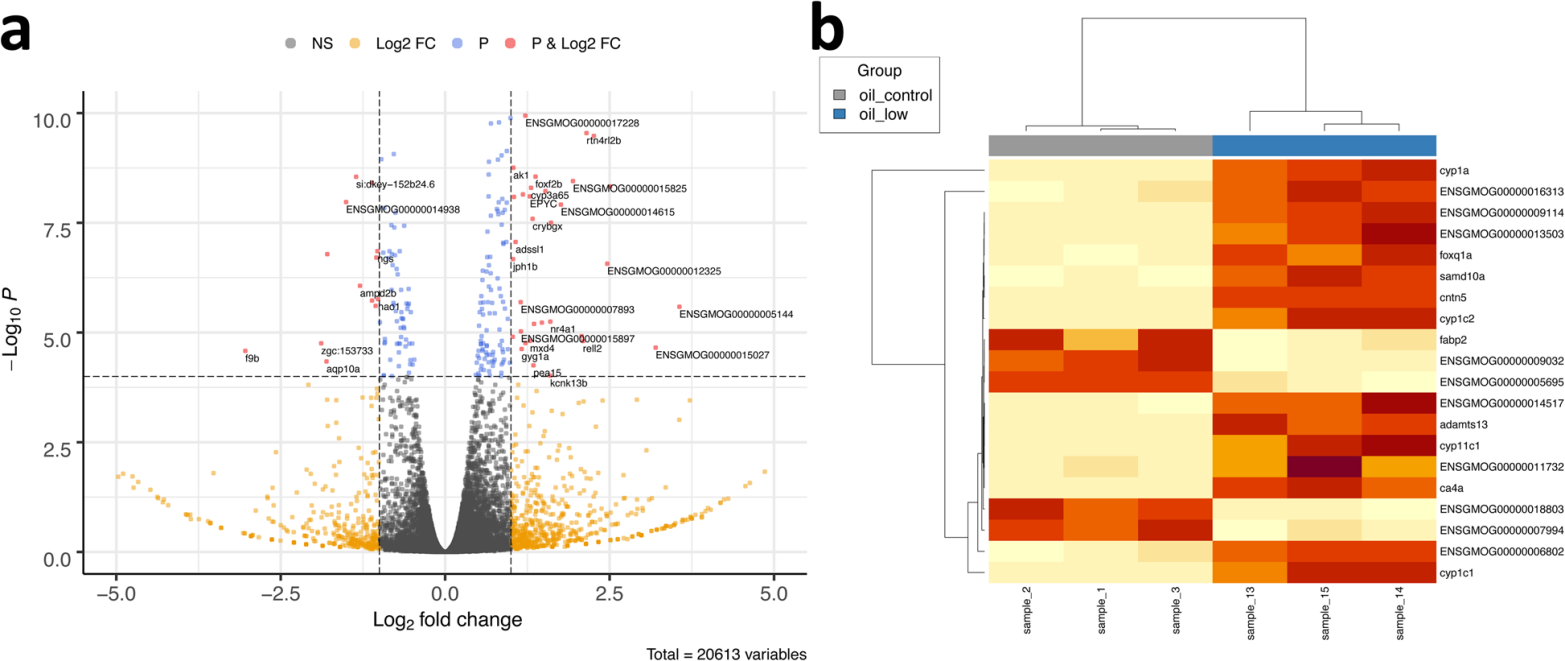
Visualization of DEA results. **a** Volcano plot with labeled genes who pass the thresholds of both Fold Change and *P*-value. **b** Hierarchical clustering heatmap with samples along the x-axis and differentially expressed genes along the y-axis

During DEA, a statistical test is performed on the raw abundance (both edgeR and DESeq2 prefer raw other than normalized abundance) tables of transcripts/genes. The result includes important statistics such as Log Fold Change, false discovery rates (FDRs) or adjusted *P*-value for each transcript/gene. With a predefined threshold of FDR (default value is 0.05), the transcripts/genes with a lower FDR are reported as significantly differentially expressed, and they are included in a second table. Besides the tables mentioned above, DEA also generates visualizations including a volcano plot (Fig. 3a) and a heatmap (Fig. 3b).

Williams et al. evaluated hundreds of combinatorial implementations of the most commonly used tools for their impact on DEA results, and they concluded that the method of differential expression analysis exhibited the strongest impact compared with the choice of tools in the other steps [40]. We have evaluated RASflow on the benchmarking dataset they generated using both the transcriptome and the genome as mapping reference, and in both cases, DESeq2 has a higher recall and edgeR has a higher precision, meaning that edgeR is more conservative in reporting a gene as differentially expressed in this study case. The differentially expressed gene list of each workflow and their performance, including values and ranks for recall and precision against the evaluated workflows in [40], can be found in Additional file 3.

### Runtime

The most time-consuming part of the whole workflow is the alignment step. As already mentioned, pseudo alignment to a transcriptome is much faster than alignment to a genome. RASflow was run on four real datasets using a 1TB RAM 60 cores Dell PowerEdge R910 machine and the runtime is shown in Table 1. RASflow was also tested on the mouse dataset using Windows Subsystem for Linux on an 8GB RAM 4 cores Intel Core 2 machine, and the runtime is shown in Table 2. As Table 1 shows, alignment using a genome as reference takes much longer than using a transcriptome, especially when the dataset is large (datasets “Cod" and “Human") or the job is run on a personal computer (dataset “Mouse_pc").

**Table 1:** Alignment runtime of three datasets

Dataset	Number of samples	Size of raw data (GB)	Runtime of alignment (HH:MM)	
			Transcriptome as reference	Genome as reference
Cod	47	244	05:32	69:18
Human	28	137	03:14	20:03
Benchmark	32	36	02:37	11:22
Mouse	8	9.3	00:28	03:46
Mouse_pc ^∗^	8	9.3	01:11	19:31

^*^This was run on a personal computer

**Table 2:** Comparison of RASflow with the other workflows published between 2017 and 2019

workflow	quality control	organism	mapping reference	workflow for DEA ^∗^	hardware requirement	installation	programming requirement	year	ref
RASflow	yes	all	genome transcriptome	GB & TB	low	easy	low	2020	NA
UTAP	yes	5	genome	GB	high	easy	low	2019	[12]
ARMOR	yes	all	genome transcriptome	TB	high	easy	low	2019	[13]
VIPER	yes	2	genome	GB	high	easy	low	2018	[14]
BioJupies	no	2	genome	GB	low	web application	low	2018	[15]
hppRNA	yes	2	genome transcriptome	GB & TB	low	medium	medium	2018	[16]
aRNApipe	yes	all	genome	GB	high	hard	high	2017	[17]
RNACocktail	no	all	genome transcriptome	GB & TB	low	hard	high	2017	[18]

^*^GB: genome based — gene/transcript quantification and DEA based on reads mapped to a genome; TB: transcriptome based

## Discussion

### Virtual environment by Conda

The whole workflow is installed and run in a virtual environment created by Conda. While creating the virtual environment, all dependencies using the specified versions are installed at once. This ensures not only the smooth installation and running of RASflow, but also a reproducible analysis independent of the operating system and machine.

### Snakemake as framework

Snakemake is a scalable workflow engine that helps to manage workflows in an easy way. It divides the whole workflow into rules with each rule accomplishing one step of the workflow. The input of one rule is the output from the rule corresponding to the previous step, making the dataflow easy to track. Thanks to this logic, the whole workflow becomes highly modular, so users can easily expand the workflow or replace part of it, also for complicated workflows.

RASflow organizes the rules carrying out one big step of the workflow in one file (with extension.rules). All the files are then integrated into one main file (main.py). For the users who are satisfied with RASflow’s default setting, they can manage the workflow simply through the configuration file to tell RASflow which pipeline and which tools they want to use. Advanced users may change the settings and parameters in the.rules files and may also substitute tools for example to try out new methods as they are published.

### Transciptome and genome as reference

RASflow allows users to supply their own genomic or transcriptomic reference. This enables users to study expression in species where no public reference is available or the users have alternative references that they wish to utilize. It should be noted that if one aims for transcript-level analysis, a transcriptome should be used as reference.

But some analyses other than DEA require the reads to be mapped to a genome and gene-level DEA is more robust and experimentally actionable, so RASflow still provides the traditional workflow of genome alignment and DEA based on gene counts.

### Comparison with other tools

We compared RASflow to other existing workflows as shown in Table 2. As we can see from the table, some workflows do not include QC steps [15, 18]. Some of the workflows are limited to specific organisms typically human or mouse and in some cases other model organisms [12, 14–16]. Some of them have functionality only for mapping reads to a reference genome and do not support the use of a transcriptome reference [12, 14, 15, 17]. ARMOR includes both genome and transcriptome as mapping reference but does not support genome-based quantification of expression and subsequent DEA.

Considering hardware requirement, BioJupies is marked as “low" because it is a web application and the compute capacity is offered on the server side. The workflows marked with “high" use STAR for genome alignment which requires about 27GB of RAM to align reads to the human genome. hppRNA and RNACocktail support both STAR and other aligners which require comparably low RAM, such as HISAT2 which is used in RASflow. Tests performed show that RASflow can be used to run human genome alignment smoothly on a personal computer with only 8GB of RAM.

As for workflow installation, RASflow, UTAP, ARMOR, and VIPER all use Conda to create a virtual environment and to install the required software, making workflow installation easy and robust. hppRNA provides scripts to automatically install all the required software but as it is not done through the use of a virtual environment, some software may conflict with software already installed on the machine. The aRNApipe and RNACocktail workflows require the user to install all the software manually which is time-consuming and can also easily lead to version conflicts.

After installation, executing the workflow can also present challenges. In order to use the aRNApipe and RNACocktail workflows on their own data, the user needs to know programming very well. The hppRNA workflow comes with a very detailed and useful manual for the user to follow which helps a lot. The UTAP and BioJupies workflows both provide graphical user interfaces and can be used without any programming skills. While the remaining workflows do not provide graphical interfaces, they use Snakemake to manage all the steps in the workflow, making them easy to use also for those with limited programming skills.

### Extension of RASflow

Thanks to the high modularity of RASflow, it is very easy to exchange the tools applied in RASflow with other tools if they are more appropriate for specific research interest or they are newly developed. Thanks to the feedback from users, we have already added the htseq-count tool for feature counting and the DESeq2 tool for DEA as extra options since the first version of RASflow. Advanced users can also do this by themselves without much effort. We welcome any feedback and contribution through GitHub page to improve RASflow.

RASflow can also be extended to realize other functions, such as Single Nucleotide Variant (SNV) detection, pathway analysis, and so on.

## Conclusions

RASflow is a light-weight and easy-to-manage RNA-Seq analysis workflow. It includes the complete workflow for RNA-Seq analysis, starting with QC of the raw FASTQ files, going through optional trimming, alignment and feature counting (if the reads are mapped to a genome), pseudo alignment (if transcriptome is used as mapping reference), gene- or transcript- level DEA, and visualization of the output from DEA.

RASflow is designed in such a way that it can be applied by a wide range of users. It requires little programming skills and a well-written tutorial helps users go through the whole workflow making it very easy to set up and run RASflow from scratch. RASflow has low hardware requirements so that it can be run on almost any personal computer. It can also be scaled up to make full use of the computing power of a supercomputer or cluster. RASflow can be applied to data of any organism and the user can choose to map the reads to a transcriptome or a genome. It also supports the use of user-supplied transcriptome or genome references.

RASflow is built on the basis of Conda and Snakemake, making installation and management very easy. All the required tools are available on the Anaconda cloud (https://anaconda.org/) and are wrapped in a virtual environment managed by Conda, making RASflow independent of the underlying system thus avoiding package/library version conflicts. The whole workflow is defined by rules managed by Snakemake, which makes it highly modular. This means that the advanced users can easily extract parts of the workflow or expand it based on their own research needs, and replace the tools used in RASflow with other tools to explore new pipelines for analyzing RNA-Seq data.

## Availability and requirements

Project name: RASflow. Project home page: https://github.com/zhxiaokang/RASflowOperating system(s): Linux, macOS and Windows.Programming language: Python, R, ShellOther requirements: CondaLicense: MIT LicenseAny restrictions to use by non-academics: N/A.

## Supplementary information

**Additional file 1** Figure S1. An overview of output folder of example data.

**Additional file 2** Figure S2. (a) The mean quality scores of raw reads from human prostate cancer data. (b) The mean quality scores of trimmed reads from human prostate cancer data.

**Additional file 3** Tables of differentially expressed gene lists of RASflow using both the transcriptome and the genome as mapping reference and using DESeq2 and edgeR as differential expression analysis methods and their performance.

## Data Availability

All the datasets and source codes are available on GitHub.

## Abbreviations

DEA: Differential expression analysis
FDR: False discovery rate
QC: Quality control
RASflow: RNA-Seq analysis Snakemake workflow
RNA-Seq: RNA sequencing
SNV: Single nucleotide variant
SRA: Sequence read archive
TMM: Trimmed mean of M values
TPM: Transcript per million
