# Supplementary material for: RASflow: an RNA-Seq analysis workflow with Snakemake
Source: BMC Bioinformatics. 2020 Mar 18;21:110. doi: 10.1186/s12859-020-3433-x (PMC7079470; doi:10.1186/s12859-020-3433-x)
Supplement: Supplementary file 1 — Additional file 1 Figure S1. An overview of output folder of example data. [file 12859_2020_3433_MOESM1_ESM.pdf]

- test
  - fastqc
  - fastqc_after_trimming
  - genome
    - alignmentQC
    - countFile
    - dea
      - countGroup
      - DEA
      - visualization
    - report_align_count_data
    - report_align_count.html
  - trans
    - dea
      - countGroup
      - DEA
        - gene-level
        - transcript-level
      - visualization
    - quant
    - report_quantify_data
    - report_quantify.html
    - tpmFile
