# Supplementary figures and images for: RASflow: an RNA-Seq analysis workflow with Snakemake
Source: BMC Bioinformatics. 2020 Mar 18;21:110. doi: 10.1186/s12859-020-3433-x (PMC7079470; doi:10.1186/s12859-020-3433-x)

**a**

FastQC: Mean Quality Scores

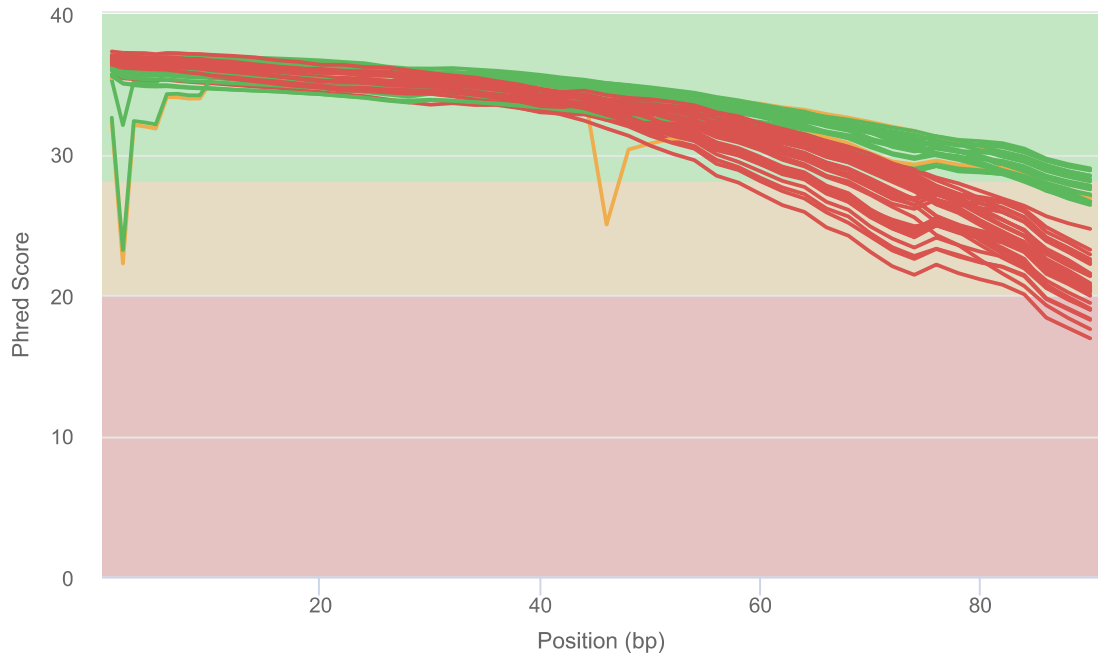**b**

FastQC: Mean Quality Scores

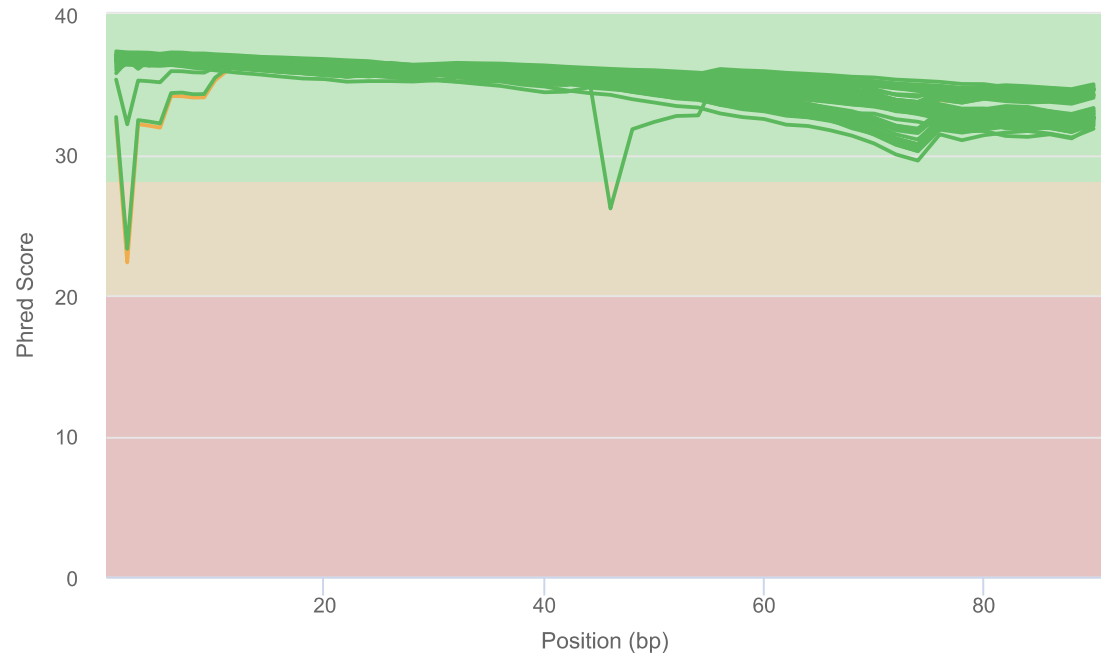

Supplement: Supplementary file 2 — Additional file 2 Figure S2. (a) The mean quality scores of raw reads from human prostate cancer data. (b) The mean quality scores of trimmed reads from human prostate cancer data. [file 12859_2020_3433_MOESM2_ESM.pdf]
